# *Clonorchis sinensis* calcium-binding protein Cs16 causes acute hepatic injury possibly by reprogramming the metabolic pathway of bone marrow-derived monocytes

**DOI:** 10.3389/fcimb.2023.1280358

**Published:** 2023-10-18

**Authors:** Qi Li, Xiao Li, Shuo Kan, Ting-Jun Zhu, Chang Li, Xin-Yue Du, Xin Wang, Hui-Bo Yan, Chen-Yun Wu, Guang-Jie Chen, Men-Bao Qian, Min Yan, Zhao-Jun Wang

**Affiliations:** ^1^ Shanghai Institute of Immunology, Department of Immunology and Microbiology, Shanghai Jiao Tong University School of Medicine, Shanghai, China; ^2^ Department of Microbiology and Immunology, Kunming Medical University, Kunming, China; ^3^ National Institute of Parasitic Diseases, Chinese Center for Disease Control and Prevention (Chinese Center for Tropical Diseases Research), World Health Organization (WHO) Collaborating Centre for Tropical Diseases, National Health Commission (NHC) Key Laboratory of Parasite and Vector Biology; National Center for International Research on Tropical Diseases, Shanghai, China; ^4^ School of Global Health, Chinese Center for Tropical Diseases Research, Shanghai Jiao Tong University School of Medicine, Shanghai, China

**Keywords:** *Clonorchis sinensis*, inflammation, metabolism, calcium binding protein, monocytes

## Abstract

**Introduction:**

*Clonorchis sinensis* infection results in various complications in the liver and biliary systems and is a neglected tropical disease in Eastern Asia. In this study, we report that *C. sinensis* calcium-binding protein Cs16 activates host immune cells and induces immunopathology in liver.

**Methods:**

Immunohistochemistry was used to detect the localization of Cs16 in *C. sinensis* adult worms. ELISA was used to detect the serum levels of anti-Cs16 IgG antibody in infected humans and mice. Bile duct injection model was used to figure out the role of Cs16 in vivo. RT-qPCR and ELISA were used to detect the cytokine production from Cs16-treated BMMs *in vitro*. Seahorse assay was used to detect the metabolic pathway of Cs16-treated BMMs in vitro.

**Result:**

Cs16 localizes in the tegument and gut of *C. sinensis*. Humans and mice with *C. sinensis* infection exhibited increased levels of anti-Cs16-specific antibody. Using the bile duct injection technique, we found that Cs16 induced obvious inflammation and hepatic necrosis *in vivo*. Cs16 treatment caused the upregulation of inflammatory cytokines in innate immune cells. Moreover, Cs16-treated monocytes relied more on the glycolytic metabolic pathway.

**Discussion:**

Our findings suggest that Cs16 is a potential pathogenic factor derived from *C. sinensis* adult worm. By reprogramming the metabolic pathway of innate immune cells, Cs16 triggers pro-inflammatory responses in the liver, and therefore, Cs16 is a potential target for the prevention and treatment of clonorchiasis.

## 1 Introduction

*Clonorchis sinensis* is one of the regionally epidemic zoonotic parasites, affecting more than 15 million people around the world, and is mainly endemic in China, South Korea, Vietnam, and part of Russia (Yoshida, 2012; Lee et al., 2018). The adult flukes living in the biliary trees of the liver induce chronic inflammation, periductal fibrosis, and even the adenomatous hyperplasia of biliary epithelial cells, all of which are closely related to the occurrence of cholangiocarcinoma (Kim et al., 2016). Therefore, *C. sinensis* was listed as a Group 1 carcinogen by the International Agency for Research on Cancer (Bouvard et al., 2009).

The pathogenesis of *C. sinensis* infection encompasses several factors, including mechanical obstruction and injury by the worms, and immunopathology caused by infection-related inflammation (van Tong et al., 2017). Inflammation in clonorchiasis can be induced by secondary bacterial infection and toxic effects of the worms’ tegumental proteins or excretory–secretory products (ESPs) (Qian et al., 2015). For example, ESPs from *C. sinensis* trigger increases in free radicals and NF-κB-mediated inflammation (Nam et al., 2012). It is a key issue to identify important molecules contributing to *C. sinensis* infection-related pathology, which might serve as drug and vaccine targets.

Elongation factor hand (EF-hand) structure proteins are ubiquitously expressed in most of helminths and bind with Ca^2+^, exerting diverse biological functions (Thomas and Timson, 2016). These calcium-binding proteins induce host immune response and specific antibody production. For example, SjTP22.4 is a calcium-binding tegumental protein of *Schistosoma japonicum.* SjTP22.4 immunization may relieve the symptom of schistosomiasis by inducing host IgG production (Zhang et al., 2012). Our previous research showed that *S. japonicum* egg specific protein SjE16.7 can recruit myeloid innate immune cells and promote the formation of inflammatory hepatic granuloma (Wu et al., 2014; Fang et al., 2015). Cs16 is a *C. sinensis* 16-kDa calcium-binding protein, which has an EF-hand motif and is a homologue of SjE16.7. In this study, we report that Cs16 serves as a pathogenic factor of *C. sinensis*. Distributed in the tegument and gut of worm, Cs16 activates host immunocytes by regulating cell metabolism and then triggers inflammation in the liver.

## 2 Materials and methods

### 2.1 Ethics statement

All animal experiments were approved by the Institutional Animal Care and Use Committee (IACUC) of Shanghai Jiao Tong University School of Medicine (project number A-2019-053, 069).

Human subject research was approved by the Ethics Committee of the National Institute of Parasitic Diseases, Chinese Center for Disease Control and Prevention in Shanghai, China (reference no. 2011-005). All individuals or their guardians for those aged <18 years have provided written informed consent.

### 2.2 Mice

Six- to eight-week-old male C57BL/6 or BALB/c mice were purchased from Shanghai Laboratory Animal Center, Chinese Academy of Sciences. Mice were housed under specific pathogen-free conditions and fed autoclaved food and water.

### 2.3 Cells

Bone marrow-derived monocytes (BMMs) were harvested from tibias and femurs of mice. Seeded on the petri dishes, cells were cultured in Dulbecco′s Modified Eagle′s Medium (DMEM, Thermo Fisher Scientific, USA) containing 10% heat-inactivated FBS, 2 mM L-glutamine, and 100 U/mL penicillin/streptomycin supplemented with 50 ng/mL M-CSF (R&D Systems, USA). Four days later, cells were dissociated with 5 mM EDTA·2Na and considered as BMMs. For further experiments, BMMs were stimulated with 5 μg/mL Cs16 or 5 μg/mL CsAWA for 0, 24, and 48 h, respectively, unless noted otherwise.

CCLP, RBE, and HuCCT1 human cholangiocarcinoma cell lines were cultured in DMEM containing 10% heat-inactivated FBS, 2 mM L-glutamine, and 100 U/mL penicillin/streptomycin.

### 2.4 *C. sinensis* infection

*C. sinensis* metacercariae were collected from *Pseudorasbora parva* from a clonorchiasis endemic area in China through the digestion method and 6-week-old BALB/c mice were infected with 50 metacercariae of *C. sinensis* by gastric gavage. Mice were then sacrificed after 28 weeks post-infection and sera were obtained for anti-CsAWA IgG and anti-Cs16 IgG antibody detection.

### 2.5 Preparation of *C. sinensis* adult worm antigen

*C. sinensis* adult worm antigen (CsAWA) was prepared as described previously (Kan et al., 2022). In brief, *C. sinensis* adult worms suspended in PBS were lysed by sonication in an ice-chilled water bath. Then, the lysed homogenate was centrifuged at 15,000 *g* for 20 min at 4°C. The supernatant was dialyzed against PBS at 4°C overnight and used as CsAWA. Protein concentration was measured by the BCA Protein Assay Kit (Sangon Biotech, China).

### 2.6 Expression and purification of recombinant Cs16

The eukaryotic expression system has been successfully established in our own lab and recombinant Cs16 from yeast expression system was prepared as described previously (PU et al., 2018). Briefly, to express the eukaryotic Cs16 *in vitro*, a pair of primers designed for target gene amplification was listed below: 5′-TCCGGAATTCCATCATCATCATCATCATATGCGATTTAGTTCACATGAGC-3′ (Forward) and 5′-TTGCGGCCGCTTATTCAGTATCTCCTGAACC-3′ (Reverse). The vector pPIC9K (Invitrogen, USA) subcloned with the Cs16 coding gene was transformed in *Pichia pastoris* strain GS115. The generated protein was fused with His-tag at the N terminal for affinity purification. The recombinant His-Cs16 collected from the yeast culture supernatant was purified by Ni-NTA Superflow Cartridges (QIAGEN, Germany) according to the manufacturer’s instructions. The molecular weight and purity of recombinant proteins were identified by SDS-PAGE. Protein concentration was measured by the BCA Protein Assay Kit. Denatured Cs16 was obtained with a 95°C, 30-min treatment.

### 2.7 Preparation of polyclonal antibody

Cs16 antiserum was prepared in C57BL/6 mice. Cs16 (50 μg) was formulated with either Freund′s complete (primary) or Freund′s incomplete (two boosts at 2-week intervals) adjuvant (Sigma-Aldrich, USA) and injected subcutaneously into mice. Mice were sacrificed 2 weeks after the final antigen immunization. Immunoglobulins from sera were first precipitated with ammonium sulfate and then purified using Protein A/G PLUS-Agarose Beads (Santa Cruz Biotechnology, USA) according to the manufacturer’s instructions.

### 2.8 Immunohistochemical assay

To determine the localization of Cs16 in *C. sinensis* adult worms, worms were fixed in 4% PFA, embedded in paraffin, and sliced at 3- to 5-μm thicknesses. Paraffin sections were deparaffinized and rehydrated, and then the heat-induced antigen retrieval method was used. Sections were blocked with 3% BSA for 45 min and then incubated with mouse anti-Cs16 polyclonal antibody diluted 1:50 in PBS overnight at 4°C. Identical concentration of control antibody from normal mouse sera was used as negative control. Sections were washed three times with PBS and then incubated with HRP-conjugated anti-mouse IgG (Cell Signaling Technology, USA) for 1 h at room temperature in the dark. After washing, sections were developed using the DAB (Diaminobenzidine) substrate system (Maxim, China). Counterstaining was done with hematoxylin. Sections were examined with an Olympus BX51 microscope (Olympus, Japan). Positive area was calculated with ImageJ software (NIH, USA).

### 2.9 Enzyme-linked immunosorbent assay

Antibody reactivity of humans or mice sera against Cs16 or CsAWA was determined by enzyme-linked immunosorbent assay (ELISA). Briefly, 96-well plates were coated with 100 μL of 100 μg/mL Cs16 or 2.5 μg/mL CsAWA overnight at 4°C. Sera were diluted 1:100 and HRP-conjugated goat anti-human IgG (Sigma-Aldrich, USA, 1:5,000 dilution) or goat anti-mouse IgG (Invitrogen, USA, 1:5,000 dilution) was used as the secondary antibody. Next, reactions were developed using 3,3′,5,5′-tetramethylbenzidine (TMB) substrates and stopped with 2 N H_2_SO_4_. The optical densities were read at 450 nm in a microwell reader system (Biotek, USA).

The levels of IL-1β, IL-6, and TNF-α in the supernatant of BMMs were measured by commercial ELISA kits (R&D Systems, USA) according to the manufacturer’s instructions.

### 2.10 Bile duct injection model

The bile duct injection technique was performed according to the protocol (Berntsen et al., 2018). In brief, 8-week-old male C57BL/6 mice were administered general anesthesia with pentobarbital sodium. For the surgery, median laparotomy was performed with a 15- to 20-mm vertical incision of the skin and peritoneum in line with the linea alba caudally from the sternum. The common bile duct (CBD) was firstly clamped. Fine-precision surgical equipment were subsequently used for the gall bladder catheterization; forceps with marked holding strength that close flat and have gently blunted tips to avoid tissue puncture were used for grasping the gall bladder. The gall bladder incision was made with the aid of sharp microscissors and to guide the catheter through the small incision using serrated, slightly curved forceps. The 20-μL fluid containing 20 μg of Cs16 or CsAWA was injected into the biliary tree. The control mice were injected with equal volume of PBS. After injection, the bulldog clamp was removed from the CBD. A loop of nonabsorbable 7-0 suture was made at the cystic bile duct directly in front of the catheter that was then removed by gentle retraction while performing a functional cholecystectomy by closing the suture completely. Approximately 50 μL of sterile physiological saline preheated to 37°C was administered in the abdominal cavity to avoid drying of the internal organs before the peritoneal and skin incisions were closed by suture and clips, respectively. Serum alanine aminotransferase (ALT) and aspartate aminotransferase (AST) were measured by Beckman-Coulter chemistry analyzer AU5800.

### 2.11 Histology and scoring

PFA-fixed liver tissues were embedded in paraffin, cut in 3- to 5-μm sections, and deparaffinized. Serial sections were stained with hematoxylin–eosin (H&E). Sections were examined with an Olympus BX51 microscope (Olympus, Japan). Mouse histological activity index (mHAI) of liver sections was scored according to the protocol (Goodman, 2007).

### 2.12 Cytometric bead array

Key serum cytokines for mouse inflammation, including IL-1β, IL-6, IL-10, TNF-α, and IFN-β were measured using a LEGENDplex™ Mouse Inflammation Panel Kit (BioLegend, USA). The data were acquired on a BD FACSCanto™ II flow cytometer, analyzed using LEGENDplex™ Data Analysis Software, and calculated by standard curves according to the manufacturer’s instructions.

### 2.13 Cell proliferation assay

Cell proliferation was quantified using Cell Counting Kit-8 (CCK-8, YEASEN, China) according to the manufacturer’s instructions. Briefly, cells were seeded into 96-well plates and incubated with 10 μL per well of the reagent for 1 h at 37°C and proliferation was quantified by measuring the absorbance at 450 nm using a microplate reader.

### 2.14 RNA isolation and real-time quantitative PCR

Total RNA was extracted from BMMs or liver tissues with the High Pure RNA Isolation Kit (Roche, Germany) in accordance with the manufacturer’s instructions. RNA quality (nucleic acid concentration, A_260/280_ and A_260/230_ ratios) was examined using a NanoDrop2000 (Thermo Fisher Scientific, USA) microvolume UV-Vis spectrophotometer. RNA (1 μg) was used for first-strand cDNA synthesis. Mouse complementary DNA (cDNA) was reverse-transcribed with the RevertAid First Strand cDNA Synthesis Kit (Invitrogen, USA) and real-time quantitative PCR was performed on a 7500 Fast Real-Time PCR System (Applied Biosystems, USA) using FastStart Universal SYBR Green Master Mix (Roche, Germany). Thermocycler conditions comprised an initial holding at 50°C for 2 min and a subsequent holding at 95°C for 10 min, which was followed by a two-step PCR program at 95°C for 15 s and 60°C for 60 s for 40 cycles. Relative gene expression was normalized to *β-actin*. Primer sequences are listed in **Supplementary Table 1**.

### 2.15 Measurement of nitric oxide production

To determine nitric oxide (NO) production, BMMs were stimulated with 5 μg/mL Cs16 or CsAWA for 48 h. NO was measured in the supernatant of the cultured cells as nitrite using an NO assay kit (Beyotime, China), according to the manufacturer’s instructions. Briefly, a standard curve was prepared with standard nitrite solutions in DMEM. The standard solutions or cell supernatants were reacted with nitrate reductase for 30 min in a 96-well plate, and then Griess reagent I and Griess reagent II were added. After incubation at room temperature for 10 min, the absorbance at 540 nm was read in a microplate reader (Thermo Fisher Scientific, USA).

### 2.16 ATP assay

BMMs seeded on the 96-well plates were lysed with ATP lysis buffer (Beyotime Biotechnology, China) on ice. Cell lysates were added to a reaction mixture containing luciferase and luciferin for bioluminescence quantification. Relative light unit (RLU) was measured by a luminometer Gen5 (Biotek, USA). ATP level was normalized to total protein content by BCA assay.

### 2.17 Oxygen consumption and extracellular acidification measurement

Extracellular acidification rate (ECAR) and oxygen consumption rate (OCR) were determined using a Seahorse XFe96 Extracellular Flux Analyzer (Agilent Technologies, USA) with commercial kits. BMMs were seeded in specialized cell culture microplates at a density of 1×10^5^ cells/well. One day prior to the measurement, a Seahorse XFe96 cartridge was loaded with 200 μL of XF Calibrant solution per well and incubated overnight at 37°C in a CO_2_-free atmosphere. One hour before the measurement, cells were resuspended with XF base medium and incubated at 37°C in a CO_2_-free atmosphere. The ports of the Seahorse cartridge were loaded with glucose, oligomycin, and 2-DG for the glycolysis stress test and oligomycin, FCCP, rotenone/antimycin A for the mitochondrial stress test. After sensor calibration, assays were run as detailed in the manufacturer’s manuals recording ECAR and OCR over time. Metabolic parameters were derived from the XF Wave software. All experiments were performed in quadruplicates. Raw values were normalized to the total protein content for each well.

### 2.18 Statistical analyses

The data were presented as mean ± s.e.m. from two or more independent experiments. Statistical analyses were performed using GraphPad Prism 8 software. Statistical significance was calculated using the unpaired, two-tailed Student’s *t*-test or one-way ANOVA followed by *post-hoc* Tukey′s test as detailed in the figure legends. *p*-values < 0.05 were considered to indicate a significant difference. Asterisks used to indicate significance correspond to the following: *p < 0.05, **p < 0.01, ***p < 0.005.

## 3 Results

### 3.1 Cs16 is expressed in *C. sinensis* and recognized by the host immune system

**Figure 1:**
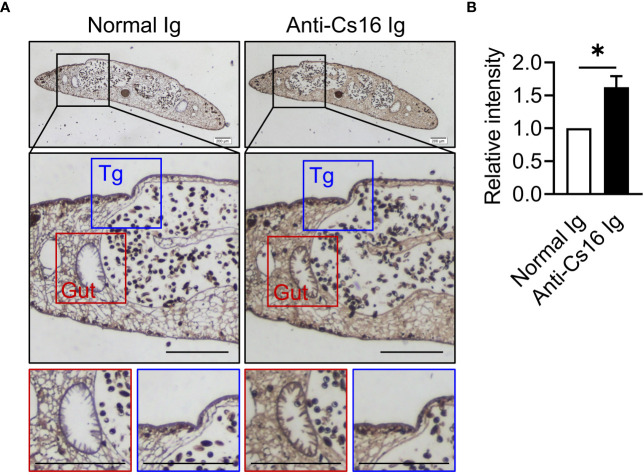
Immunolocalization of Cs16 in adult *C. sinensis*. **(A)** The representative immunohistochemical images of Cs16 in *C. sinensis* adult worms. Left panel: normal Ig (negative control) as primary antibody. Right panel: anti-Cs16 Ig as primary antibody. Scale bar, 200 μm. Tg, tegument. **(B)** The relative intensity of positive area. The data were shown as the mean ± s.e.m., *p < 0.05, unpaired two-tailed Student’s *t*-test.

To identify the characteristics of Cs16, we prepared recombinant Cs16 from *P. pastoris* and anti-Cs16-specific antibodies from mice. Then, we probed the sites of Cs16 expression within adult *C. sinensis.* Cs16 exhibited ubiquitous expression through all tissues, particularly in the gut and tegument of adult worms (**Figure 1**), suggesting that it could be involved in the interaction between worms and hosts.

**Figure 2:**
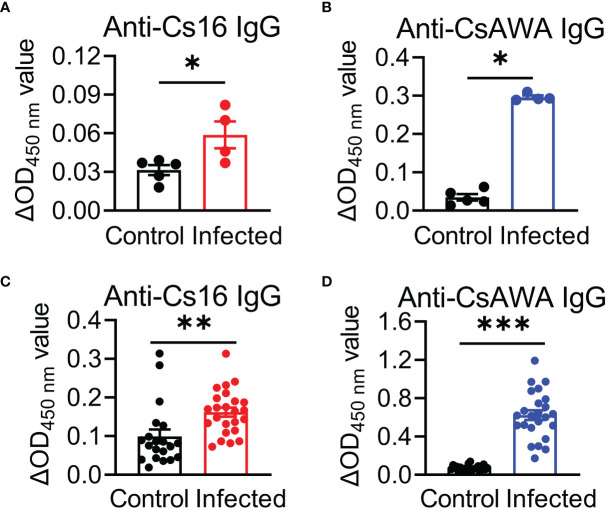
Serum levels of anti-Cs16 and anti-CsAWA IgG in *C*. *sinensis*-infected mice and human. **(A, B)** Serum anti-Cs16 and anti-CsAWA IgG levels of control mice (*n* = 5) and *C*. *sinensis*-infected mice (*n* = 4). **(C, D)** Serum anti-Cs16 and anti-CsAWA IgG levels of human with healthy control (*n* = 23) and *C*. *sinensis*-infected people (*n* = 24). CsAWA, *C*. *sinensis* adult worm antigen. The data were shown as the mean ± s.e.m., *p < 0.05, **p < 0.01, ***p < 0.005, unpaired two-tailed Student’s *t*-test.

We then asked the antigenicity of Cs16, whether this protein provokes immune responses during infection with *C. sinensis*. To address this question, mice were infected with *C. sinensis* metacercariae. The antibody production against *C. sinensis* adult extract (CsAWA) and Cs16 was detected by ELISA. The ELISA results showed that the infected mice developed specific antibodies against Cs16 as CsAWA, while sera from control mice without infection revealed no specific antibodies (**Figures 2A, B**). Similar phenotypes were observed in *C. sinensis*-infected people (**Figures 2C, D**), implying that Cs16 can be recognized by the host immune system and induce immune responses.

### 3.2 Cs16 results in inflammation and acute hepatic injury *in vivo*

**Figure 3:**
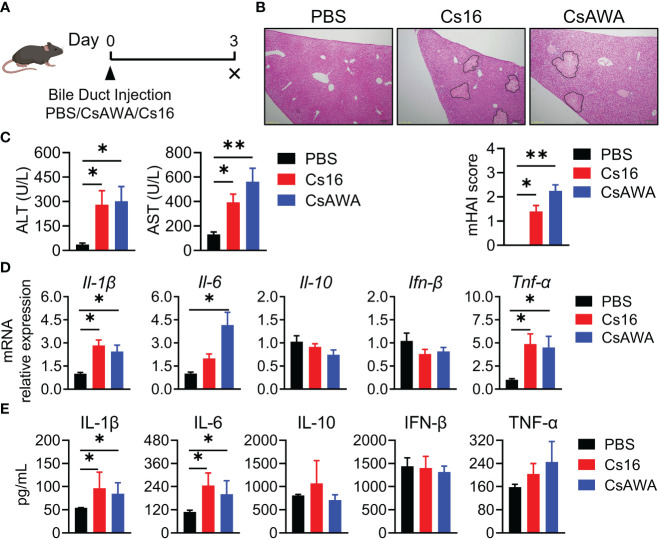
Pathology of bile duct injection with Cs16 and CsAWA. **(A)** Schematic diagram of the bile duct injection technique. **(B)** Representative images of liver tissues after Cs16 and CsAWA injection (upper panel) and mouse hepatic activity index (mHAI, lower panel). **(C)** Serum levels of ALT (left panel) and AST (right panel) in mice injected with PBS, Cs16, and CsAWA for 72 h. **(D)** Transcript levels of *Il-1β*, *Il-6*, *Il-10*, *Ifn-β*, and *Tnf-α* in the liver of mice injected with PBS, Cs16, or CsAWA for 72 h. **(E)** Serum levels of IL-1β, IL-6, IL-10, IFN-β, and TNF-α in mice injected with PBS, Cs16, or CsAWA for 72 h. CsAWA, *C*. *sinensis* adult worm antigen. ALT, alanine aminotransferase. AST, aspartate aminotransferase. The data were shown as the mean ± s.e.m., PBS, *n* = 5; Cs16, *n* = 5; CsAWA, *n* = 4. *p < 0.05, **p < 0.01, one-way ANOVA followed by *post-hoc* Tukey’s test.

Since *C. sinensis* adults live in bile ducts, we used bile duct injection technique to explore the role of Cs16 *in vivo* (**Figure 3A**). Results showed that liver tissues of both Cs16-treated and CsAWA-treated mice exhibited more inflammatory cell infiltration and severe necrosis of liver parenchyma than PBS-treated mice (**Figure 3B**). Meanwhile, serum ALT and AST levels, which reflect the extent of liver damage, were significantly increased after Cs16 and CsAWA treatment (**Figure 3C**). Elevated inflammatory cytokines secreted by inflammatory cells, such as *Il-1β*, *Il-6*, and *Tnf-α*, were also observed in liver tissues of both Cs16-treated and CsAWA-treated mice (**Figure 3D**). Meanwhile, serum IL-1β and IL-6 levels were increased after Cs16 or CsAWA injected into the bile ducts of mice (**Figure 3E**). The above results indicated that Cs16 can promote inflammation and hepatic injury *in vivo*.

### 3.3 Cs16 promotes inflammatory cytokine secretion of BMMs

**Figure 4:**
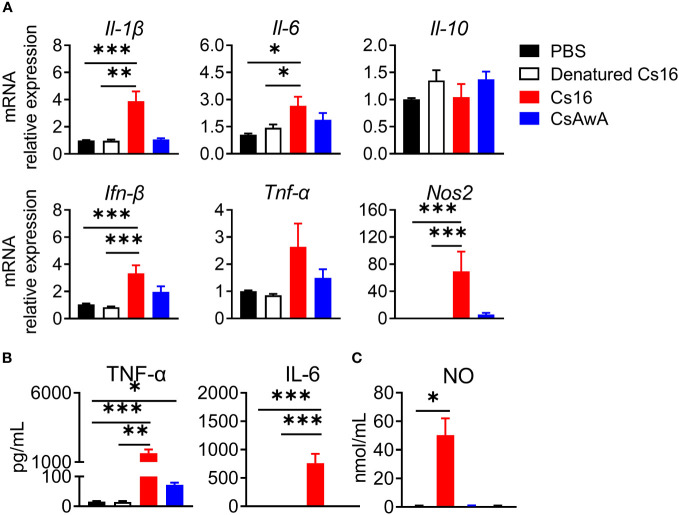
Inflammatory cytokine levels in the supernatant of BMMs after Cs16 and CsAWA treatment. **(A)** Transcript levels of *Il-1β*, *Il-6*, *Il-10*, *Ifn-β*, *Tnf-α*, and *Nos2* in BMMs treated with Cs16 or CsAWA for 48 h. **(B)** TNF-α (left panel) and IL-6 (right panel) levels in the supernatant of BMMs treated with Cs16 or CsAWA for 48 h. **(C)** NO levels in the supernatant of BMMs treated with Cs16 or CsAWA for 48 h. CsAWA, *C*. *sinensis* adult worm antigen. The data were representative of two or three independent experiments (biological replicates) and shown as the mean ± s.e.m., *p < 0.05, **p <0.01, ***p < 0.005, one-way ANOVA followed by *post-hoc* Tukey’s test.

We then test the roles of Cs16 *in vitro*. Cs16 has no cytotoxicity under the concentration of 5 μg/mL, and the proliferation of cholangiocytes and immunocytes was not influenced (**Supplementary Figures 1**, **2**). Given the pro-inflammatory property of SjE16.7 and the phylogenetic similarity between SjE16.7 and Cs16, we detected the effects of Cs16 on inflammatory cytokine production. BMMs were challenged with Cs16. Enhanced inflammatory cytokine release such as *Il-1β*, *Il-6*, *Ifn-β*, and *Nos2*, an inflammatory enzyme, in transcriptional levels was found in cells treated with Cs16 for 48 h. Similarly, TNF-α, IL-6, and NO levels in the supernatant of the cultured cells were increased after Cs16 treatment (**Figure 4**). These results indicated that Cs16 activates BMMs and induces a release of pro-inflammatory cytokines.

### 3.4 Cs16-treated BMMs prefer glycolysis for energy generation

**Figure 5:**
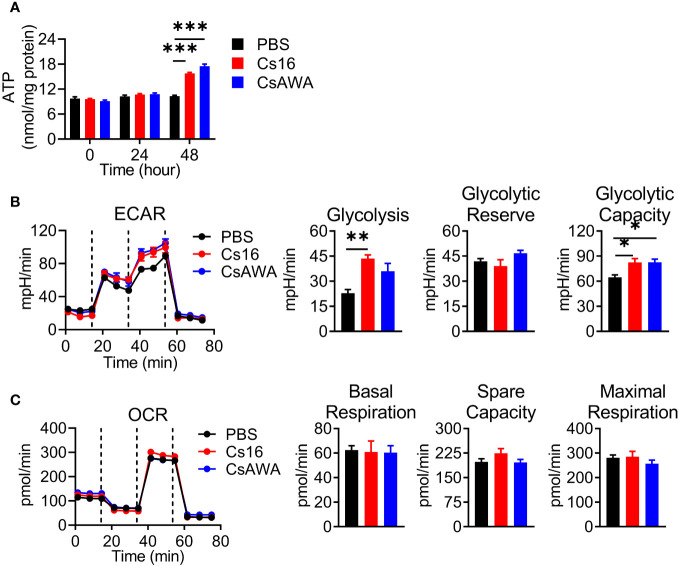
Cellular ATP, ECAR, and OCR levels after Cs16 and CsAWA treatment. **(A)** ATP levels in BMMs treated with Cs16 or CsAWA for 48 h. **(B)** Dynamic curves of ECAR (left panel), glycolysis, glycolytic reserve, and glycolytic capacity (right panel) in BMMs treated with Cs16 or CsAWA for 48 h. **(C)** Dynamic curves of OCR (left panel), basal respiration, spare capacity, and maximal respiration (right panel) in BMMs treated with Cs16 or CsAWA for 48 h. CsAWA, *C*. *sinensis* adult worm antigen. The data were representative of two or three independent experiments (biological replicates) and shown as the mean ± s.e.m., *p < 0.05, **p < 0.01, ***p < 0.005, one-way ANOVA followed by *post-hoc* Tukey’s test.

Immune cell activation favors the ATP production. We detected the effects of Cs16 on cellular ATP levels. The results showed that BMMs stimulated with Cs16 or CsAWA produced more ATP than controls (**Figure 5A**). It is well known that the main source of ATP derives from glycolysis or oxidative phosphorylation. Therefore, we assessed the ECAR and OCR of cells treated with Cs16 and CsAWA. Results showed that ECAR was significantly increased after Cs16 or CsAWA stimulation (**Figure 5B**); meanwhile, there was no difference between control and antigen stimulation in OCR (**Figure 5C**). The above results suggested that BMMs stimulated with Cs16 relied more on glycolysis for energy metabolism.

## 4 Discussion

Clonorchiasis is one of the most important neglected tropical diseases in Eastern Asia (Lai et al., 2016). *C. sinensis* adults, which dwell in bile ducts, cause hepatic and biliary disease with biliary epithelial hyperplasia, periductal fibrosis, and cystic changes in the ducts and even facilitate the development of cholangiocarcinoma (Na et al., 2020). However, the component of *C. sinensis* that plays a role in the pathogenesis is not well explored. As a *C. sinensis* adult worm-derived protein, Cs16 can induce host immune responses. This conclusion is based on the following observations: (a) Cs16 localizes in the tegument and gut of *C. sinensis* adult worms; (b) the levels of anti-Cs16 IgG antibody are increased in both humans and mice with *C. sinensis* infection; (c) Cs16 activates BMMs and promotes inflammatory cytokines release; and (d) Cs16 induces acute hepatic injury *in vivo*.

Cs16 protein has low molecular weight and a conserved calcium-binding motif called the EF-hand, which is similar to S100 protein families in humans (Sreejit et al., 2020). Several S100 proteins are considered as damage-associated molecular pattern molecules (DAMPs), and associated with infection, cellular stress, tissue damage, and cancer (Lim et al., 2011). It has been reported that S100 proteins induce inflammatory responses during bacteria and virus infection (Tsai et al., 2014; Zackular et al., 2015; Kozlyuk et al., 2019). Bai et al. found that inhibition of S100A9 alleviates acute liver injury, which is accompanied by M2-like macrophages activation (Bai et al., 2021). In our study, we observed Cs16 promotes glycolytic pathway (a characteristic of M1-like macrophages) and acute liver injury, indicating the pro-inflammatory role of Cs16. Host immune responses after *C. sinensis* infection is a dynamic process. Kim et al. reported that hepatic macrophages preferentially differentiated into M1 macrophages during the early stages of *C. sinensis* infection, but the numbers of M2 macrophages increased during the fibrotic and cirrhotic stage of infection (Kim et al., 2017). It is of interest to investigate the roles of Cs16 in chronic infection.

In this study, we used the bile duct injection model and examined inflammatory cytokines after antigen treatment for 72 h, which is usually considered to be in the acute immune response stage (4–96 h post-infection or stimulation). Monocytes and macrophages are key components of the innate immune system and are involved in the development of acute inflammatory disorders. In addition, it has been reported that monocytes play important roles in cholangitis (Kunzmann et al., 2020). In this study, we found that Cs16 activates BMMs and induces inflammatory cytokine production *in vitro*; thus, we propose that Cs16 causes acute hepatic injury by activating BMMs. Besides monocytes, Cs16 may also activate other cells and contribute to inflammation. It needs to be further studied. Excretory–secretory proteins (ESPs) of liver flukes are critical for the pathogenesis, nutrient metabolism, etiology, and immune response of liver cancer. Our data showed that Cs16 localizes in the gut and tegument of *C. sinensis* adults and anti-Cs16 IgG is detected in infected mouse or human sera, suggesting that Cs16 may secrete from adults and be recognized by the host. However, Shi et al. identified the ESPs of *C. sinensis* after culture for 6 h, and results showed that there exist some kind of calcium-binding proteins, in which Cs16 is not listed (Shi et al., 2020). It might be due to the fact that ESPs obtained from the *in vitro* culture of *C. sinensis* adult may not fully mimic the true microenvironment *in vivo*. The appropriate method to check whether Cs16 belongs to ESPs or not is worth exploring in the future.

**Figure 6:**
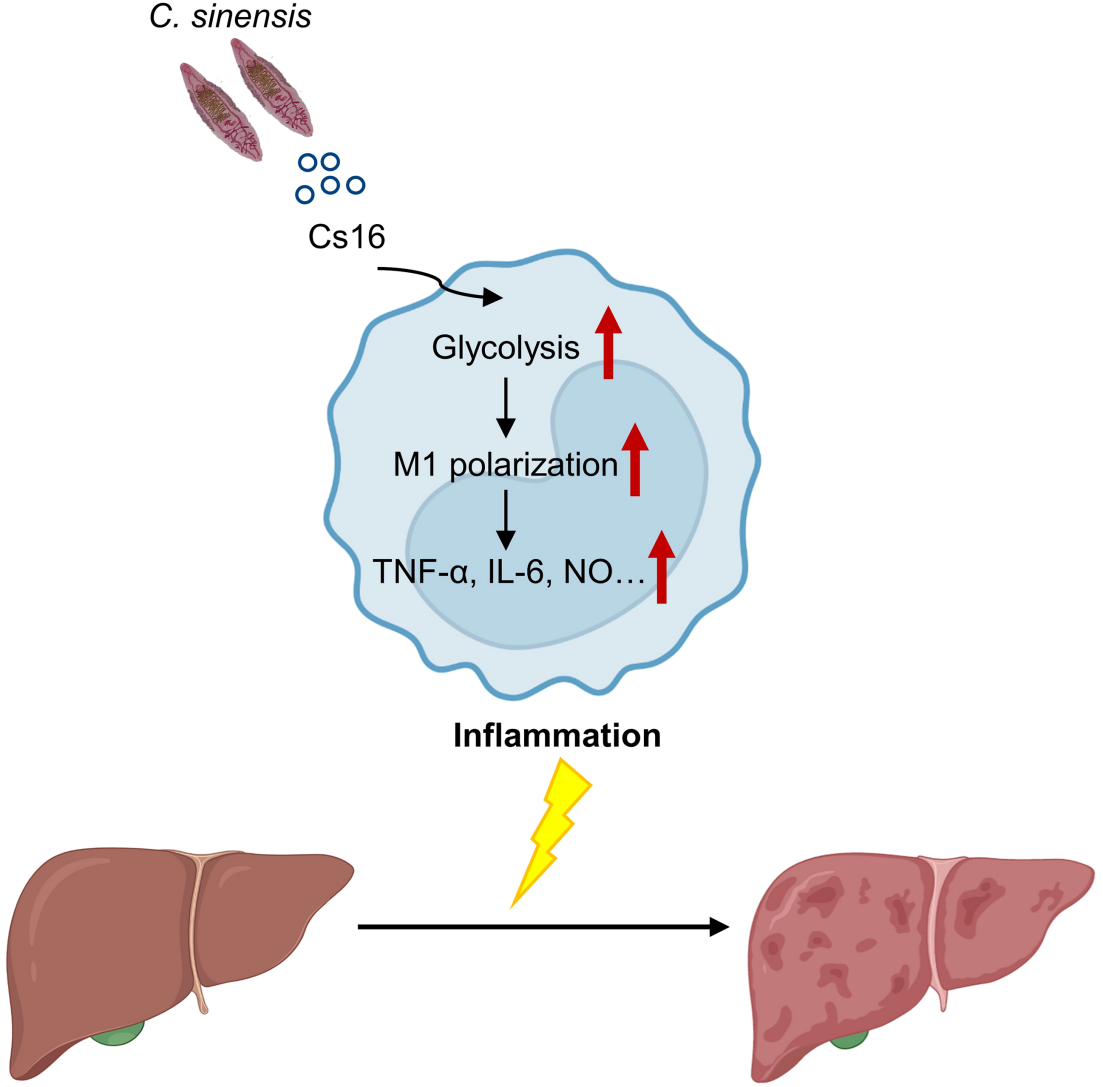
A proposed working model for Cs16 in regulating the metabolic pathway of BMMs and promoting hepatic injury.

Calcium-binding proteins are a heterogeneous and wide group of proteins that participate in numerous cellular functions (Yanez et al., 2012). Chung et al. reported that CsCa8 was derived from *C. sinensis* adult worms and possessed an EF-hand structure, which localized in the oral and ventral suckers of the adult worm (Chung et al., 2013). Kim et al. reported a 21.6-kDa tegumental protein from *C. sinensis* (Kim et al., 2012). Both of them are calcium-binding proteins, but their effects on host immune systems are not well elucidated. Cs16 derived from a *C. sinensis* adult can be recognized by hosts, and then induces pro-inflammatory immune responses via altering metabolic pathways of immunocytes. We reported that Cs16 can activate host immune cells and induces hepatic injury, indicating that Cs16 is a potential pathogenic factor of *C. sinensis* and can be a target for diagnosis and treatment of clonorchiasis (**Figure 6**).

## Data availability statement

The original contributions presented in the study are included in the article/**Supplementary Material**. Further inquiries can be directed to the corresponding authors.

## Ethics statement

The studies involving humans were approved by Ethics Committee of the National Institute of Parasitic Diseases, Chinese Center for Disease Control and Prevention in Shanghai, China (reference no.2011-005). All individuals or their guardians for those aged <18 years have provided written informed consent. The studies were conducted in accordance with the local legislation and institutional requirements. Written informed consent for participation in this study was provided by the participants’ legal guardians/next of kin. The animal study was approved by the Institutional Animal Care and Use Committee (IACUC) of Shanghai Jiao Tong University School of Medicine (project number A-2019-053, 069). The study was conducted in accordance with the local legislation and institutional requirements.

## Author contributions

QL: Data curation, Investigation, Methodology, Writing – original draft. XL: Data curation, Formal Analysis, Investigation, Writing – review & editing, Methodology. SK: Investigation, Writing – review & editing. T-JZ: Writing – review & editing, Resources. CL: Writing – review & editing, Investigation. X-YD: Investigation, Writing – review & editing. XW: Methodology, Writing - review & editing. H-BY: Methodology, Writing - review & editing. C-YW: Methodology, Writing – review & editing, Resources. G-JC: Supervision, Writing – review & editing. M-BQ: Resources, Supervision, Writing – review & editing, Methodology, Project administration. MY: Supervision, Writing – review & editing, Resources. Z-JW: Conceptualization, Funding acquisition, Project administration, Resources, Supervision, Writing – review & editing, Methodology.

## Supplementary material

Supplementary Figure 1 Growth curves of BMMs treated with Cs16 and CsAWA measured by CCK-8 assay. The data were representative of two or three independent experiments (biological replicates) and shown as the mean ± s.e.m.

Supplementary Figure 2 Growth curves of CCLP, RBE, HuCCT1 cells treated with Cs16 and CsAWA measured by CCK-8 assay. The data were representative of two or three independent experiments (biological replicates) and shown as the mean ± s.e.m.

Supplementary Table 1 Information for qPCR primers.
