# Supplementary figures and images for: Clonorchis sinensis calcium-binding protein Cs16 causes acute hepatic injury possibly by reprogramming the metabolic pathway of bone marrow-derived monocytes
Source: Front Cell Infect Microbiol. 2023 Oct 18;13:1280358. doi: 10.3389/fcimb.2023.1280358 (PMC10619745; doi:10.3389/fcimb.2023.1280358)

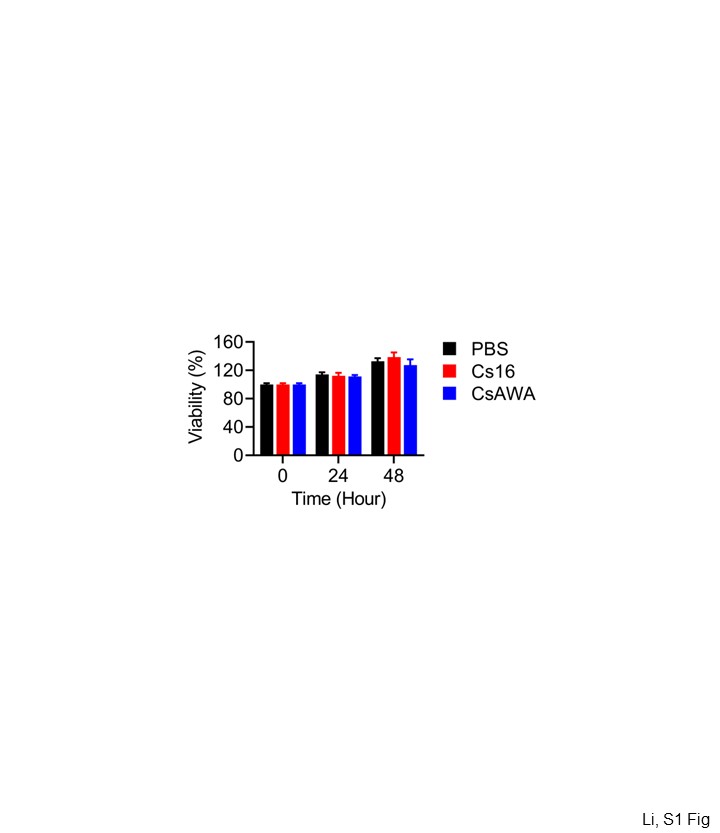

Supplement: Supplementary Figure 1 — Growth curves of BMMs treated with Cs16 and CsAWA measured by CCK-8 assay. The data were representative of two or three independent experiments (biological replicates) and shown as the mean ± s.e.m. [file Image_1.jpeg]

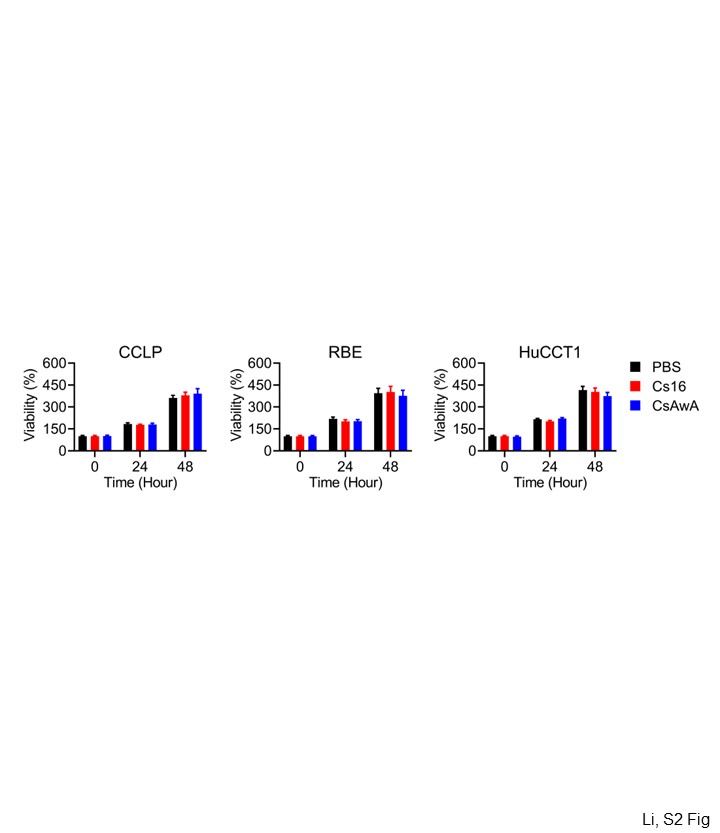

Supplement: Supplementary Figure 2 — Growth curves of CCLP, RBE, HuCCT1 cells treated with Cs16 and CsAWA measured by CCK-8 assay. The data were representative of two or three independent experiments (biological replicates) and shown as the mean ± s.e.m. [file Image_2.jpeg]

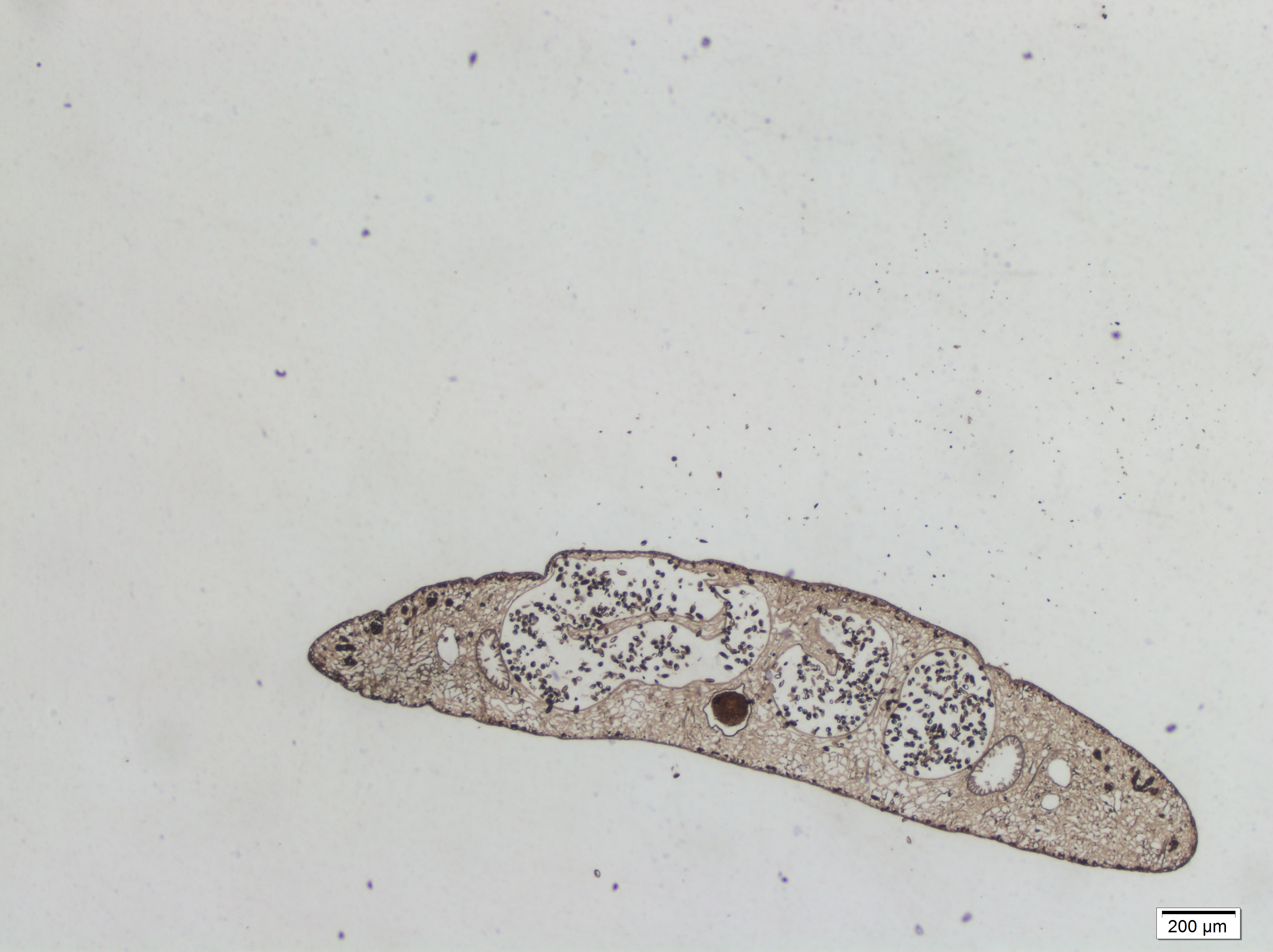

Supplement: Supplementary file 5 [file Image_3.jpeg]

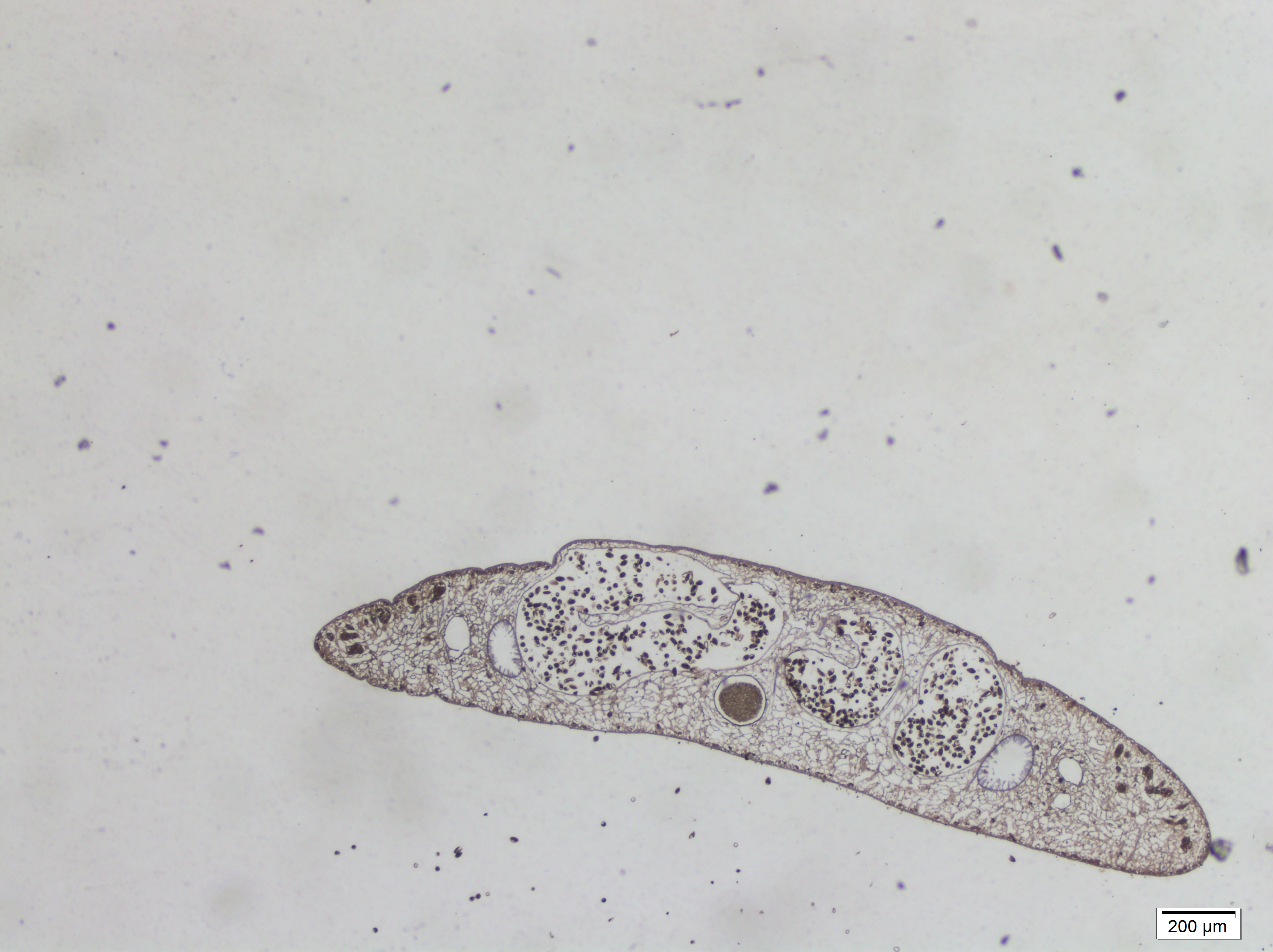

Supplement: Supplementary file 6 [file Image_4.jpeg]

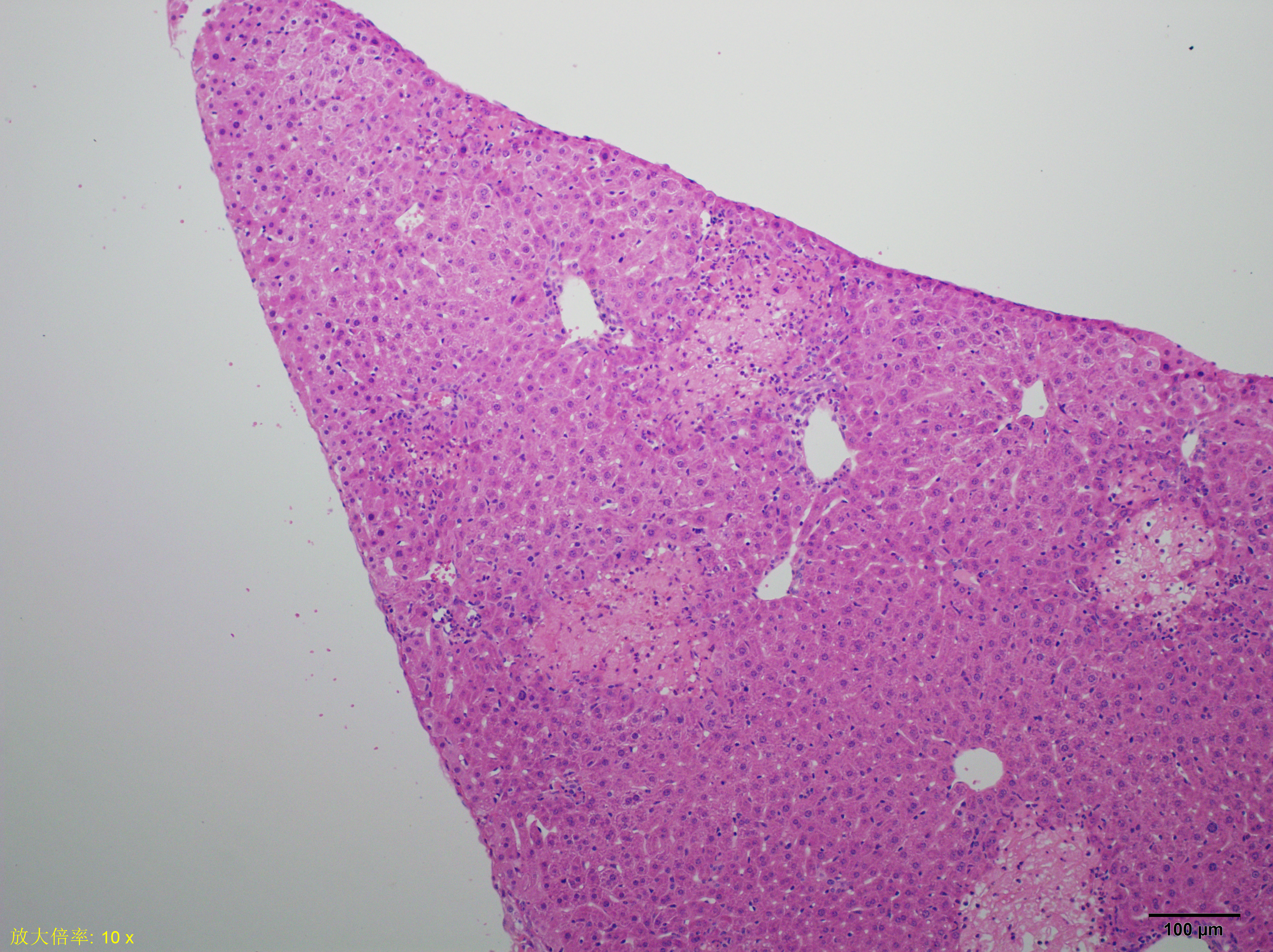

Supplement: Supplementary file 7 [file Image_5.jpeg]

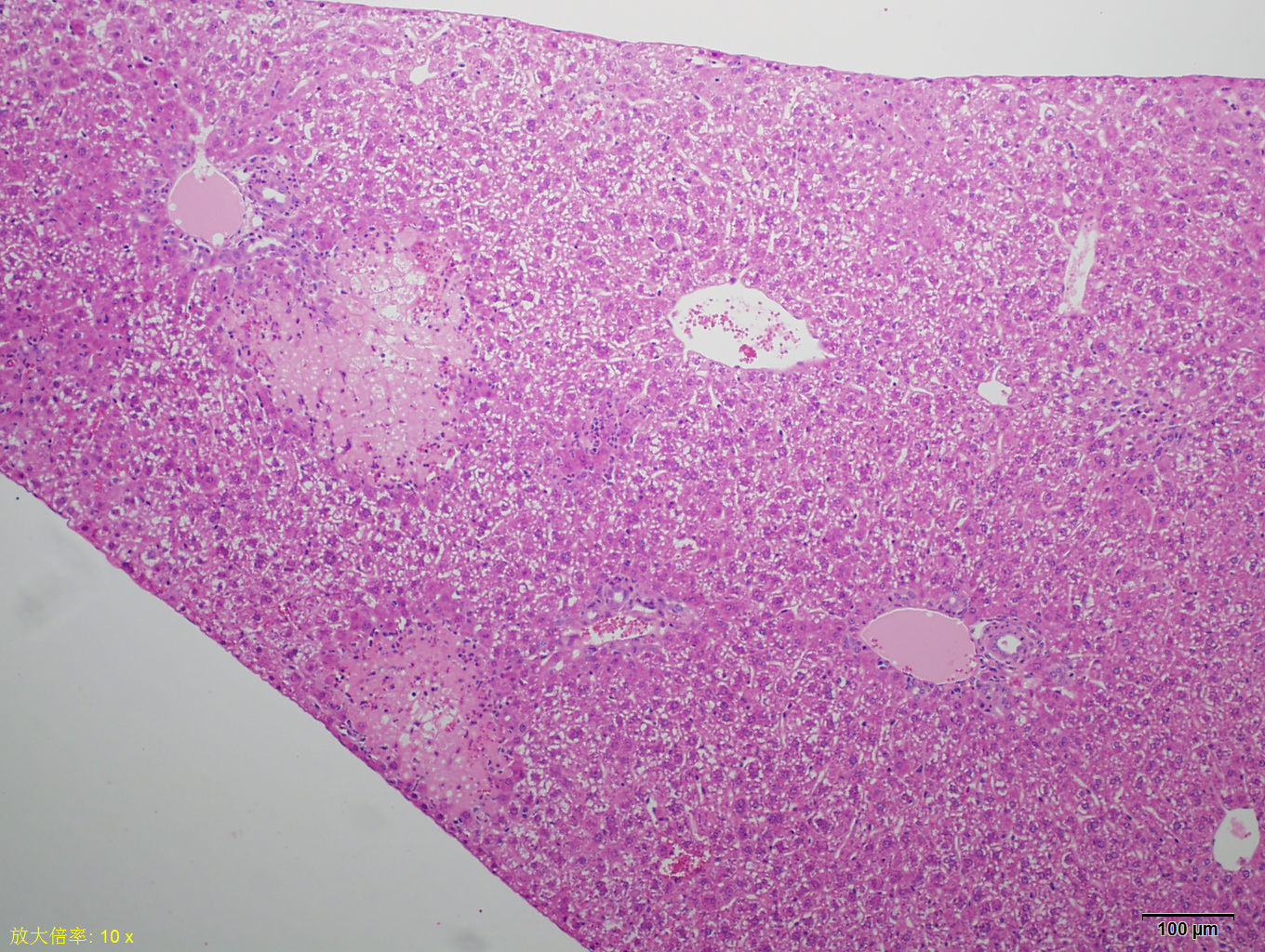

Supplement: Supplementary file 8 [file Image_6.jpeg]

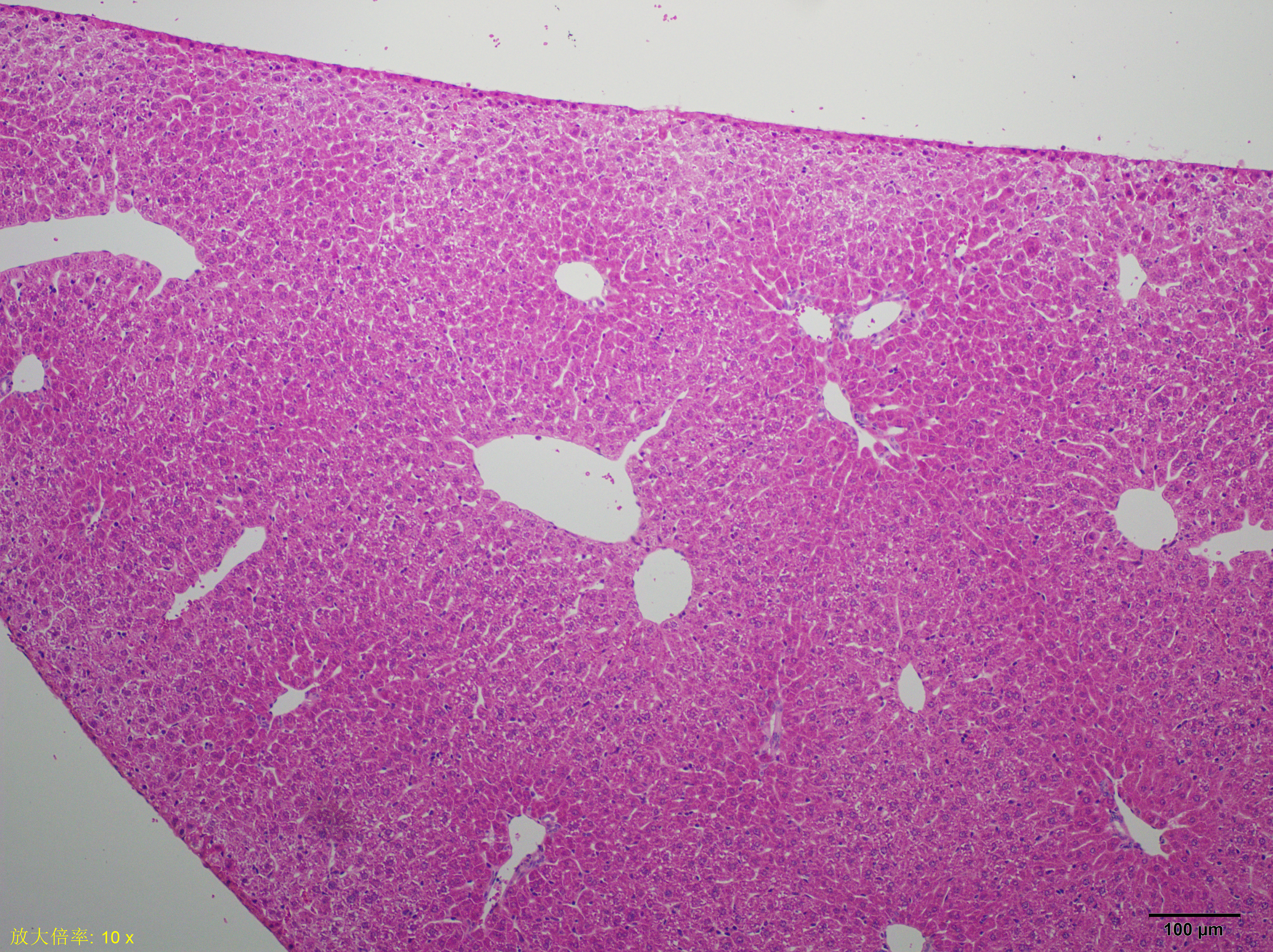

Supplement: Supplementary file 9 [file Image_7.jpeg]
